# A theoretical model for graft punch size calculations to prevent Descemet’s membrane folds following deep anterior lamellar keratoplasty

**DOI:** 10.5935/0004-2749.20200077

**Published:** 2024-02-11

**Authors:** Selim Genc, Fehim Esen, Emre Guler, Hanefi Çakir

**Affiliations:** 1 Ophthalmology Clinic, University of Health Sciences Beyoglu Eye Training and Research Hospital, Istanbul, Turkey; 2 Department of Ophthalmology, Istanbul Medeniyet University School of Medicine, Istanbul, Turkey; 3 Turkiye Hospital, Ophthalmology Clinic, Istanbul, Turkey

**Keywords:** Descemet’s membrane, Keratoconus, Keratoplasty, penetrating, Corneal topography, Cornea/pathology, Membrana de Descemet, Ceratocone, Ceratoplastia penetrante, Topografia da córnea, Córnea/patologia

## Abstract

**Purpose:**

The length of Descemet’s membrane and donor graft sizes in deep anterior lamellar keratoplasty do not match in very steep corneas, which can lead to Descemet’s membrane folds. The aim of this study is to establish a theoretical model for graft size calculations for deep anterior lamellar keratoplasty and evaluate its efficacy for preventing Descemet’s membrane folds.

**Methods:**

We calculated the arc diameter of the recipient bed by using the cosine formula and developed a table to aid surgeons in donor punch size selection. To test the usefulness of this formula, we evaluated the development of Descemet’s membrane folds in keratoconus patients with very steep corneas (K >60 D). In group 1, deep anterior lamellar keratoplasty surgeries were performed using graft sizes that were determined based on our model (n=31). In group 2, graft sizes were determined based on the empirical judgment of the surgeon without any formal calculation (n=30).

**Results:**

Our theoretical calculations demonstrated that the diameter of donor punch sizes needed to prevent Descemet’s membrane fold increases when the cornea is steeper, or the trephine size is larger. We tested the efficacy of this model on the clinical outcome of deep anterior lamellar keratoplasty. The mean age (28.9 ± 10.1 years vs. 32.8 ± 8.3 years, p=0.11) and preoperative K1 (59.2 ± 9.3 D vs. 58.1 ± 9.4 D, p=0.67), K2 (66.2 ± 6.0 D vs. 65.7 ± 7.4 D, p=0.81), and Km values (62.1 ± 7.7 D vs. 61.8 ± 8.1 D, p=0.88) were similar between the two groups. Three patients developed Descemet’s membrane folds in group 2, and none of the patients developed Descemet’s membrane folds in group 1. These results supported our theoretical calculations.

**Conclusion:**

Adjustment of donor graft size based on the calculated arc diameter of the recipient bed reduced the development of Descemet’s membrane folds after deep anterior lamellar keratoplasty in steep corneas.

## Abstract (pt)

**Objetivo:**

O comprimento da membrana de Descemet e o tamanho do enxerto doador na ceratoplastia lamelar anterior profunda não coincidem em córneas muito íngremes, o que pode levar às dobras da membrana de Descemet. O objetivo deste estudo é estabelecer um modelo teórico para cálculo do tamanho do enxerto para ceratoplastia lamelar anterior profunda e avaliar a sua eficácia na prevenção de dobras da membrana de Descemet.

**Métodos:**

Calculamos o diâmetro do arco do leito receptor usando a fórmula do cosseno e desenvolvemos uma tabela para auxiliar os cirurgiões na seleção do tamanho da punção no doador. Para testar a utilidade dessa fórmula, avaliamos o desenvolvimento das dobras da membrana de Descemet em pacientes com ceratocone com córneas muito íngremes (K>60D). No grupo 1, foram realizadas cirurgias de ceratoplastia lamelar anterior profunda, utilizando tamanhos de enxerto que foram determinados com base em nosso modelo (n=31). No grupo 2, os tamanhos dos enxertos foram determinados com base no julgamento empírico do cirurgião sem qualquer cálculo formal (n=30).

**Resultados:**

Nossos cálculos teóricos demonstraram que o diâmetro dos tamanhos da punção do doador necessários para evitar as dobras na membrana de Descemet aumenta quando a córnea é mais íngreme ou o tamanho da trefina é maior. Testamos a eficácia deste modelo no resultado clínico da ceratoplastia lamelar anterior profunda. A média de idade (28,9 ± 10,1 anos vs. 32,8 ± 8,3 anos, p=0,11) e K1 pré-operatório (59,2 ± 9,3 D vs. 58,1 ± 9,4 D, p=0,67), K2 (66,2 ± 6,0 D vs. 65,7 ± 7,4) D, p=0,81) e Km (62,1 ± 7,7 D vs. 61,8 ± 8,1 D, p=0,88) foram semelhantes entre os dois grupos. Três pacientes desenvolveram dobras na membrana de Descemet no grupo 2, e nenhum dos pacientes desenvolveu dobras na membrana de Descemet no grupo 1. Estes resultados apoiam nossos cálculos teóricos.

**Conclusão:**

O ajuste do tamanho do enxerto doador com base no diâmetro do arco calculado do leito receptor reduziu o desenvolvimento das dobras na membrana de Descemet após ceratoplastia lamelar anterior profunda em córneas íngremes.

## INTRODUCTION

Keratoplasty has been successfully used for the treatment of various corneal pathologies, including keratoconus, for many years. Three types of keratoplasty procedures have been performed for corneal stromal pathologies: penetrating keratoplasty (PK), epikeratophakia^(1)^, and deep anterior lamellar keratoplasty (DALK). For most patients, DALK is regarded as the best techni que for the management of keratoconus^(2)^. The main advantages of DALK are the absence of endothelial rejection (the endothelium of the recipient is protected) and a reduced requirement for graft quality (e.g., stored grafts may be applied)^(3,4)^. Additionally, DALK avoids many complications associated with the open-sky condition during corneal transplantation, including anterior synechiae formation, expulsive hemorrhage, and endophthalmitis^(5)^.

**Figure 1:**
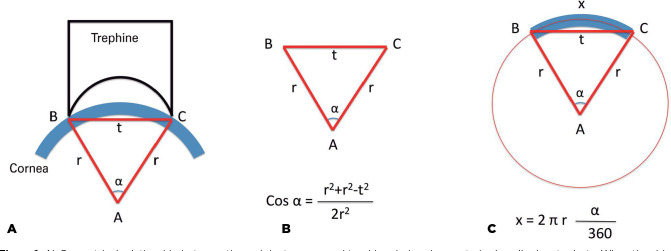
A) Geometrical relationship between the recipient cornea and trephine during deep anterior lamellar keratoplasty. When the sides of the base of the punch are connected to the center of the corneal curvature, an isosceles triangle is formed, with the radius of the corneal curvature representing the equal sides. The apical angle of this triangle corresponds to the angle that faces the arc length of the remaining Descemet’s membrane. B) Because the lengths of each side of the triangle are known, the cosine formula can be used to determine cos _α_. Inverse cosine calculation can be used to calculate the apical angle. C) The apical angle can be further used to calculate the size of the remaining arch of the Descemet’s membrane (x) on the recipient bed.

In patients with advanced keratoconus, the central corneal curvature changes dramatically and becomes very steep, with a low radius of curvature at the corneal apex. However, the donor cornea is healthy and has a normal base curve. When removing a corneal button from this very steep cornea, an arc with a curvature is removed, and the remaining Descemet’s membrane (DM) has a similar shape. The length of the arc is not equal to the diameter of the trephine applied (Figure 1). However, when graft tissue is taken from the donor cornea, suction is applied, and the cornea is nearly flat. Under these conditions, the length of the graft tissue is similar to the size of the donor punch. Therefore, when using the same size trephine (which does not flatten the recipient cornea during the incision) and donor punches (which flatten the donor corneal tissue during the incision), the diameters of the remaining recipient DM and donor tissues are not equal. This mismatch can induce DM fold formation, despite the completion of successful, uncomplicated DALK surgery in such steep corneas^(6,7)^. These folds can change the optical properties of the cornea and interfere with the quality of vision, particularly when they are located within the visual axis. Thus, the prevention of this complication is important.

The cosine formula can be used to calculate the angle of a triangle when the lengths of the sides of the triangle are known: cos α = (b^2^ + c^2^ - a^2^)/2bc. The angle can then be calculated using the inverse cosine function. When the angle that corresponds to the arc angle of the corneal button of the recipient bed is known, the length of the corneal arc can be calculated using the following formula: arc length = 2πr (α/360). This arc length is equal to the length of the remaining DM layer on the recipient bed. After the length of the remaining DM is calculated, corneal graft buttons of equal size can be prepared to match the length of the DM layer and prevent DM fold formation, which can improve the optical outcome of DALK. In the first part of this study, we used this theoretical approach to estimate the optimal donor corneal button size to fit the length of DM on the recipient bed. In the second part of this study, we evaluated the efficacy of this model for the prevention of DM folds after DALK in keratoconus patients with very steep corneas.

## METHODS

In the first part of the study, we designed a theoretical model to represent the diameters of removed tissue and DM during trephination. The cosine formula was used to calculate cos α based on different corneal curvature values and trephine sizes. The inverse cosine function was used to calculate the angle α. Subsequently, the real arc length of the corneal tissue that corresponded to the angle α was calculated, and this value represented the arc length/diameter of the corneal button removed from the recipient cornea, as well as the diameter of the remaining DM area. Because donor tissue is flattened during the incision, we assumed that the diameter of the donor tissue would be equal to the donor punch size.

In the second part of this study, we retrospectively assessed the clinical outcome of our model. We evaluated 61 eyes of 60 patients who underwent DALK with the diagnosis of keratoconus between 2010 and 2015. The selected patients were divided into two groups: in group 1, the donor corneal punch sizes were calculated based on the above-mentioned formula, and in group 2, the donor corneal punch sizes were selected arbitrarily based on the clinical judgment of the surgeon. The diagnosis of keratoconus was based on slit-lamp findings (e.g., stromal thinning, Fleischer ring, and Vogt striae) and corneal topography. All included patients had advanced keratoconus, characterized by keratometry values >60 diopters (D) in at least one meridian, according to Scheimpflug imaging (Sirius, CSO, Italy); poor spectacle-corrected visual acuity; and intolerance to rigid gas-permeable contact lenses or inappropriate contact lens fit. Patients were excluded if they exhibited other ocular pathologies, such as Fuchs’ endothelial dystrophy, cataracts, retinal disorders, and/or glaucoma. Patients were also excluded if they exhibited acute hydrops or had intraoperative complications such as unsuccessful big bubble formation or DM perforation. This study was conducted in accordance with the tenets of the Declaration of Helsinki, and the study protocol was approved by the clinical research ethics board of Istanbul Medeniyet University Goztepe Teaching and Research Hospital.

All patients underwent a detailed ophthalmological examination, including evaluation of best-corrected visual acuity (BCVA, logMAR) using the Snellen chart, slit-lamp examination, dilated fundus examination, and corneal topography using the Sirius imaging system. All DALK surgeries were performed by the same surgeon (SG) under retrobulbar anesthesia. Ninety percent of the recipient cornea (approximately 375 µm) was incised using a Barron trephine. A 5-mL syringe with a 30-gauge needle was filled with air, which was then injected into the deep stroma to form a big bubble between the corneal stroma and the DM. After big bubble formation, the posterior stroma was removed using a blunt-tipped scissor, and the DM was exposed. Barron donor punch was used to punch out donor buttons from the endothelial side, and the DM with endothelial layer was gently dissected from the button for use in endothelial keratoplasty for another patient. The donor corneal button was sutured to the recipient bed with a 10-0 nylon suture with 16 interrupted sutures. The postoperative treatment regimen included topical moxifloxacin four times per day for 1 month and topical dexamethasone 0.1% four times per day for 2-3 months. Sutures were removed at least 6 months postoperatively under the guidance of corneal topography to reduce postoperative astigmatism. Removal of the sutures was completed by 18 months postoperatively. In patients with suture loosening or vascularization of the host cornea, sutures were removed earlier.

Statistical analyses were performed using SPSS statistical software (version 15, SPSS Inc., Chicago, IL, USA). The normality of the data distribution was determined using the Kolmogorov-Smirnov test. Continuous variables were compared between groups using Student’s t-test and the Mann-Whitney U test. Categorical variables were compared between groups using Fisher’s exact test. P values <0.05 were considered statistically significant.

## RESULTS

Our theoretical modeling demonstrated that, as the cornea became steeper, the arc diameter increased in the tissue removed from the cornea. This mismatch between trephine size and arc diameter of the tissue became more apparent when the trephine size was larger, or cornea was steeper (>50 D). Our model demonstrated that, if the same size trephine and donor punches were used during surgery, the arc diameters of the DM and the graft corneal tissues were likely to be different. Because the diameter of the Descemet’s tissue is larger in steep corneas, the DM would likely fold if a smaller graft tissue were sutured on top of it. To prevent this clinical problem, we used our model to calculate DM arc sizes (or suggested donor punch sizes) for different corneal curvatures and trephine sizes (Table 1).

**Table 1:** Suggested donor punch sizes for specific corneal curvature and trephine size values

Curvature of recipient cornea (D)	Trephine size (mm)								
	6.00	6.25	6.50	6.75	7.00	7.25	7.50	7.75	8.00
45	6.17	6.45	6.72	7.00	7.28	7.57	7.85	8.14	8.44
46	6.18	6.46	6.73	7.01	7.30	7.58	7.87	8.16	8.46
47	6.19	6.47	6.75	7.03	7.31	7.6	7.89	8.19	8.49
48	6.20	6.48	6.76	7.04	7.33	7.62	7.91	8.21	8.51
49	6.21	6.49	6.77	7.05	7.34	7.63	7.93	8.23	8.54
50	6.22	6.50	6.78	7.07	7.36	7.65	7.95	8.25	8.56
51	6.23	6.51	6.79	7.08	7.38	7.67	7.97	8.28	8.59
52	6.24	6.52	6.81	7.10	7.39	7.69	8.00	8.31	8.62
53	6.25	6.53	6.82	7.11	7.41	7.71	8.02	8.33	8.65
54	6.26	6.54	6.84	7.13	7.43	7.73	8.04	8.36	8.68
55	6.27	6.56	6.85	7.15	7.45	7.76	8.07	8.39	8.71
56	6.28	6.57	6.87	7.16	7.47	7.78	8.09	8.42	8.75
57	6.29	6.58	6.88	7.18	7.49	7.8	8.12	8.45	8.78
58	6.30	6.60	6.90	7.20	7.51	7.83	8.15	8.48	8.82
59	6.32	6.61	6.91	7.22	7.53	7.85	8.18	8.51	8.86
60	6.33	6.63	6.93	7.24	7.56	7.88	8.21	8.55	8.90
61	6.34	6.64	6.95	7.26	7.58	7.91	8.24	8.59	8.94
62	6.35	6.66	6.97	7.28	7.60	7.93	8.27	8.62	8.99
63	6.37	6.67	6.98	7.30	7.63	7.96	8.31	8.66	9.03
64	6.38	6.69	7.00	7.32	7.65	7.99	8.34	8.71	9.08
65	6.40	6.71	7.02	7.35	7.68	8.03	8.38	8.75	9.13
66	6.41	6.72	7.04	7.37	7.71	8.06	8.42	8.79	9.19
67	6.43	6.74	7.06	7.40	7.74	8.09	8.46	8.84	9.24
68	6.44	6.76	7.09	7.42	7.77	8.13	8.50	8.89	9.30
69	6.46	6.78	7.11	7.45	7.80	8.17	8.55	8.95	9.37
70	6.48	6.80	7.13	7.48	7.83	8.20	8.59	9.00	9.43

In the second part of our study, we compared the clinical outcomes of surgeries performed using this model (group 1) and surgeries performed before the development of this model (group 2). The sex distribution (19 women, 12 men vs. 15 women, 15 men; p=0.44) and mean age (28.9 ± 10.1 years vs. 32.8 ± 8.3 years, p=0.1) were similar between groups 1 and 2. The mean preoperative keratometry values were 62.1 ± 7.70 D (range, 47.0 to 74.0 D) in group 1 and 61.8 ± 8.1 D (range, 48.0 to 72.0 D) in group 2 (p=0.88). The clinical characteristics of the patients are summarized in table 2.

**Table 2:** Clinical characteristics of keratoconus patients in groups 1 and 2.

	Group 1 (n=31)	Group 2 (n=30)	P
Age, years (mean ± SD)	28.9 ± 10.1	32.8 ± 8.3	0.11
Sex, female/male (n)	19/12	14/15	0.31
K1 (D ± SD)	59.2 ± 9.3	58.1 ± 9.4	0.67
K2 (D ± SD)	66.2 ± 6.0	65.7 ± 7.4	0.81
Km (D ± SD)	62.1 ± 7.7	61.8 ± 8.1	0.88
Trephine size, mm (mean ± SD)	7.5 ± 0.16	7.6 ± 0.19	0.46
Donor punch size, mm (mean ± SD)	7.8 ± 0.16	7.8 ± 0.20	0.69
Difference between punch and trephine size, mm (mean ± SD)	0.25 ± 0.00	0.24 ± 0.20	0.78
Descemet’s membrane folds (n)	0/31	3/30	0.11

SD= standard deviation.

**Figure 2:**
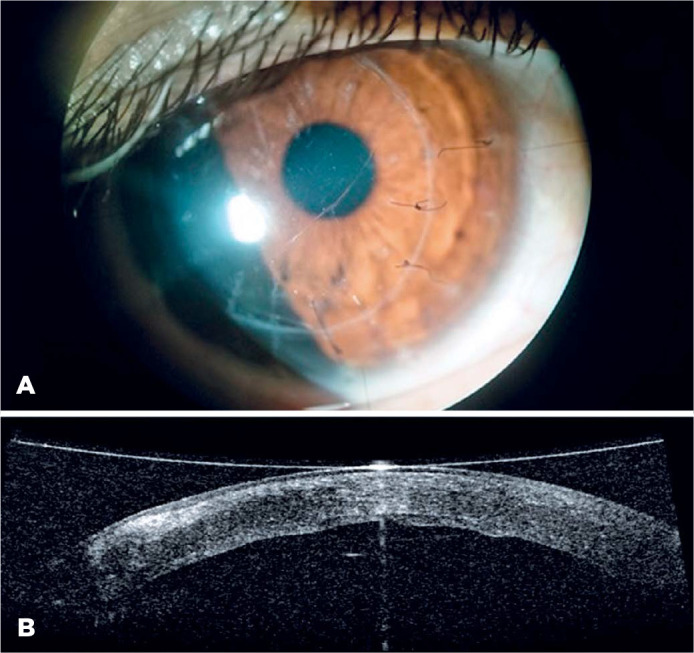
A) Slit-lamp image of a keratoconus patient who developed Descemet’s membrane (DM) folds following deep anterior lamellar keratoplasty. B) Postoperative optical coherence tomography image of DM folds.

In both groups, all operations were successful, and none of the patients experienced recipient corneal perforation. During the follow-up period, the grafts were clear in all eyes and adhered well to the recipient corneal bed. None of the patients in group 1 had DM folds, whereas DM folds occurred in three eyes at the center of the graft in group 2 (p=0.11) (Figure 2). In those three eyes, graft sizes were equal to trephine sizes, and these had been selected with the aim of flattening the cornea and reducing the magnitude of myopia induced by keratoconus. There were no other serious postoperative complications such as intraocular pressure elevation, immune rejection, or keratoconus recurrence.

## DISCUSSION

Keratoplasty has been successfully performed for the treatment of advanced-stage keratoconus^(8)^. Three types of keratoplasty are currently available for the management of keratoconus, including PK, epikeratophakia, and DALK. PK provides rapid improvement in visual acuity, but graft survival in PK depends on the quality of the graft, particularly the endothelium layer. Furthermore, the rejection rate after PK is higher (approximately 10%), which is a severe complication of this surgery^(9)^. The main limitation of epikeratophakia is that it can only be performed in patients with early stages of keratoconus, where keratometry values are <55 D^(10)^. This is relatively rare among keratoplasty candidates with keratoconus.

DALK is the preferred technique for patients with advanced keratoconus who do not have pre-existing endothelial pathology. DALK provides a clear cornea in the absence of the risk of endothelial rejection, and it is an extraocular procedure and prevents complications related to the open-sky condition during PK. Despite these benefits, DALK has three important disadvantages. First, the improvement in visual outcome is lower than that produced by PK, and this is influenced by surgical instruments and surgical experience. Second, the risk of DM perforation is only present in DALK. Third, postoperative DM folds may occur in the recipient cornea of patients with very steep corneas, and this can degrade the quality of vision (Figure 2).

Shi et al.^(7)^ reported the use of a modified DALK technique to prevent DM folds in 65 eyes with advanced keratoconus (keratometry >60 D). They dissected the cornea manually and maintained 2-mm diameters of DM at the cone apex. They used an 8-mm diameter corneal graft with a gradual pressure technique to reduce the risk of DM folds at the center of the cornea. Postoperatively, no patients had DM folds, and all grafts were successfully adhered to the recipient bed. Kakshoor et al.^(6)^ also reported the use of a modified DALK technique in patients with advanced keratoconus with steep keratometry. In their technique, bare DM was maintained in the 5 mm center, along with peripheral preservation of the posterior stromal layer. Postoperatively, no patients exhibited DM folds.

A previous study suggested that a donor trephine ≥0.25 mm larger than the recipient trephine constitutes the best anatomical match^(11)^. In addition, the use of an undersized donor may lead to some problems: the closure of the wound is more challenging and may require strict suturing, which leads to a flatter graft. Importantly, a flat graft may lead to deficient tear film distribution, as well as epithelialization defects, post-keratoplasty hyperopia, and inadequate contact lens fitting^(12)^. DALK prevents the development of wound leakage compared to PK in an undersized donor^(13)^. However, more severe DM folds may occur when an undersized graft is applied. These folds may be eliminated if the donor button is 0.5 mm larger than the recipient trephine, as reported by Fogla and Padmanabhan^(14)^.

In this study, we only included patients with steep keratometry (>60 D at least in one meridian) and excluded patients with intraoperative DM perforation. We presume that the postoperative DM folds are a result of mismatched DM and graft size, particularly in patients with very steep corneas. In our patients, DM folds did not occur when a properly calculated graft size was used (i.e., larger than the recipient trephine size). We used the cosine and arc length formulas to calculate the optimal graft size to prevent DM folds. DM folds occurred in three patients in group 2. In these patients, recipient bed and graft sizes did not follow the guidance of the above-described formulas (i.e., 7.75 mm trephine and 7.75 mm punch sizes were used). These results confirm the importance of using proper trephine and donor corneal punch sizes in patients with steep corneas in order to prevent DM folds following DALK.

In a previous study, the cosine formula was used for the calculation of aortic arc^(15)^. In our study, we used this formula to improve the outcome of ocular surgery. The length of the DM that has a circular shape was calculated using the same formula in order to determine the ideal size for a punched donor cornea (Table 1). However, trephines were only available in 0.25-mm steps, and we chose the closest size when the calculated size was not available.

To the best of our knowledge, this study is the first to demonstrate that the cosine formula can be used for the modeling of ideal graft size selection in DALK surgery and that it can successfully prevent the formation of DM folds in steep corneas. The application of this formula and the diagram provided in this article may be useful in improving the visual outcome of DALK surgery.
